# Benzothiadiazole and B-Aminobutyricacid Induce Resistance to *Ectropis Obliqua* in Tea Plants (*Camellia Sinensis* (L.) O. Kuntz)

**DOI:** 10.3390/molecules23061290

**Published:** 2018-05-28

**Authors:** Huan Li, Ying Yu, Zhenzhen Li, Emmanuel Arkorful, Yiyang Yang, Xinqiu Liu, Xinghui Li, Ronglin Li

**Affiliations:** 1 Institute of Leisure Agriculture, Jiangsu Academy of Agricultural Science, Nanjing 210014, China; muzili0822@163.com (H.L.); cbslizhenzhen@126.com (Z.L.); yangyiyang_yyy@126.com (Y.Y.); 2 Tea Research Institute, Nanjing Agricultural University, Nanjing 210095, China; 2017204032@njau.edu.cn (Y.Y.); emmamidnite@hotmail.com (E.A.); liuxinqiu@njau.edu.cn (X.L.)

**Keywords:** benzothiadiazole, β-aminobutyric acid, polyphenol oxidase, phenylalanine ammonia-lyase, *Ectropis obliqua*, induced resistance

## Abstract

In order to investigate the effect of benzothiadiazole (BTH) and β-aminobutyric acid (BABA) on the resistance of tea plants *(Camellia sinensis*) to tea geometrid (*Ectropis obliqua*), three levels each of benzothiadiazole (BTH) and β-aminobutyric acid (BABA) were sprayed on 10-year-old tea plants. Generally PPO and PAL activities increased with low concentrations of BTH and BABA treatments. Quantitative RT-PCR revealed a 1.43 and 2.72-fold increase in PPO gene expression, and 3.26 and 3.99-fold increase in PAL gene expression with 75 mg/L BTH and 400 mg/L BABA respectively. Analysis of hydrolysis of synthetic substrates also revealed that chymotrypsin-like enzyme activity present in larval midgut extracts was not significantly inhibited by BTH and BABA. However, proteinase activity was found to be inversely proportional to the age of tea geometrid. Larvae pupation rate decreased by 8.10, 10.81 and 21.62% when tea geometrid were fed with leaves treated with 25, 50 and 75 mg/L BTH solutions, while 100, 200 and 400 mg/L BABA solutions decreased same by 8.10, 16.21 and 13.51% respectively. Also, larvae development period delayed to 23.33 and 26.33 days with 75 mg/L BTH and 400 mg/L BABA treatments respectively. The results in this study; therefore, suggest that benzothiadiazole (BTH) and β-aminobutyric acid (BABA) play a role in inducing resistance in tea plants to tea geometrid, with the optimal effect achieved at BTH-3 (75 mg/L) and BABA-3 (400 mg/L), respectively.

## 1. Introduction

Tea (*Camellia sinensis*) is a popular plant which originated in the southwest of China. Currently, tea is cultivated in more than hundred countries worldwide [1].Leaves of tea plants are brewed into tea which is the most popular non-alcoholic beverage in the world. Tea cultivation in China is faced with several biotic stressors, of which tea geometrid (*Ectropis obliqua*) is the most significant [2]. Tea geometrid feeds on tea shoots and causes serious hindrances to tea production and quality, resulting in huge economic losses [3]. Several methods have been developed to control tea geometrid, yet pesticide use has been the primary technique [4]. However, pesticide use may result in severe ecological and environmental issues. In addition, residues from pesticides also pose serious health hazards to humans [5]. It is imperative to explore new effective and environmentally friendly techniques for the control of tea geometrid.

In the early 20th century several studies revealed that special chemicals known as priming agents or elicitors can induce plants’ resistance to diseases [6,7]. Usually elicitors do not inhibit pathogens directly, but rather confer to plants the ability to resist pathogens, a mechanism known as acquired immunity or systemic acquired resistance (SAR). Not only do elicitors induce plant resistance to pathogens, but also to herbivores. Most widely used elicitors include salicylic acid (SA), jasmonic acid (JA), benzothiadiazole (BTH) and β-aminobutyric acid (BABA) [8]. Wakeil reported that foliar application of jasmonic acid on leaves of wheat restrained the development of the insect pests [9]. It is also reported that HrPNEa could induce the *Myzus persica* resistance signal transportation in *Arabidopsis thaliana* [10]. Similarly, JA was found to induce the defense-related enzymes activities in cotton seedlings, and significantly inhibited the relative growth rate of *Helicoverpa armigera* [11].Series of studies conducted on the insect resistance ability of tea plants, reported that JA can induce tea plants’ resistance to *Ectropis oblique* [12,13] . Also treatment of tea plants with SA resulted in increased probing number and significant decrease in ingestion duration of tea leaf hopper *Empoasca onukii* [14]. Xin et al. also reported that (*Z*)-3-hexenol treatment increased the polyphenol oxidase (PPO) activity and volatile production in tea plants. The induced tea plants reduced the performance of tea geometrid and became highly attractive to the main parasitoid wasp, *Apanteles glomeratus* [2].

According to the literature, the upregulation of PPO and POD, as well as the accumulation of polyphenols may be disadvantageous to herbivores. Several studies have reported that PPO and POD as well as PAL are involved in induced plant resistance to herbivores, and elicitors which induce the activity of these enzymes could; therefore, enhance plants resistance to herbivores [6,15,16]. The action way of the leaf enzymes and phenols in tea plants to herbivores may, in some extent, be similar to that in pathogens except for the digestive system, where the mid-gut enzymes may be affected by the leaf constituent [17,18]. In our previous study it was found that BABA and BTH could induce the up-regulation of PAL and PPO as well as the polyphenol accumulation in tea plants [1]. It could; therefore, be speculated that BABA and BTH may play a critical role in inducing tea plant’s resistance to herbivores and pathogens. Studies have shown that BABA and BTH induce disease resistance, and; therefore, it is necessary to explore the possibility of these elicitors to induce insect resistance ability to tea plants. On this account, the present study was conducted to investigate the biochemical changes in leaves of tea plants treated with BABA and BTH, and the role these elicitors play in inducing resistance in tea plants to *Ectropis obliqua*. The result in this study will contribute to the exploration of the possibility of developing a new way to control *Ectropis obliqua* based on the principle of systemic acquired resistance.

## 2. Results

### 2.1. Differential Expression Analysis of PPO and PAL Genes

Total RNA extracted from the treated samples was identified using agarose gel electrophoresis. Two clear bands (28S rRNA and 18S rRNA) were identified upon agarose gel electrophoresis analysis and the ratio of A260/A280 was between 1.90 and 2.0 without trailing. This; therefore, suggests that the extracted RNA was of good quality and met the demand for PCR analysis.

**Figure 1:**
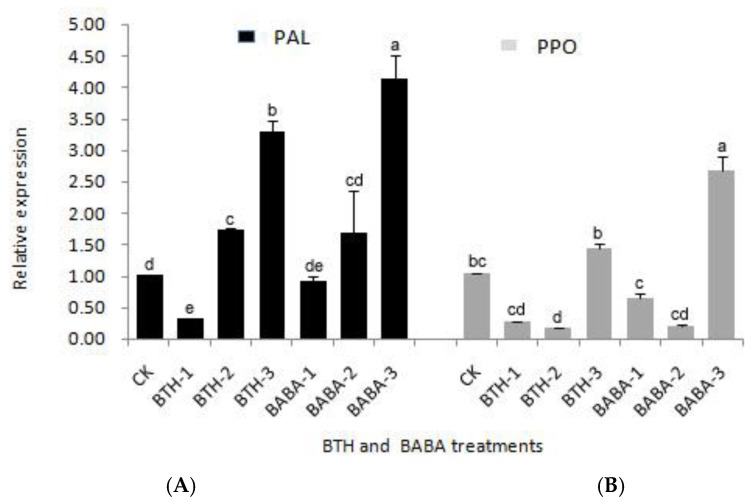
Relative Expression of PAL and PPO genes in different treatments. (**A**) Relative expression of PAL gene in BTH and BABA treatments. (**B**) Relative expression of PPO gene in BTH and BABA treatments. Note: BTH-1 (25 mg/L), BTH-2 (50 mg/L), BTH-3 (75 mg/L); BABA-1 (100 mg/L), BABA-2 (200 mg/L), BABA-3 (400 mg/L). Data are presented as Means ± SD. The error bars represent the standard deviation. Different lowercase letters depict significant differences.

Quantitative real-time PCR analysis also revealed the standard ratios of PAL and PPO (Figure 1). Obviously, the expression levels of PAL gene in the tea leaves of BTH-2, BTH-3, and BABA-2, BABA-3 treatment groups were higher than that of the control. The order of PAL gene expression levels in BTH and BABA treatment groups was observed as BTH-3 > BTH-2 > BTH-1, and BABA-3 > BABA-2 > BABA-1 respectively (Figure 1A). There was; therefore, a significant increase in PAL gene expression in BTH-3 and BABA-3 treatments with 3.26 and 3.99 times increase in gene expression as compared with the control, respectively (Figure 1A). This; therefore, suggests that high concentration of elicitors treatment is more effective in adjusting the PAL gene expression.

On the contrary, an irregular pattern of PPO gene expression was observed in both BTH and BABA treatment groups (Figure 1). It was observed that the higher the elicitor treatment concentration, the lesser the PPO gene expression, compared with the control treatments, and this was same for both BTH and BABA treatment groups (Figure 1B). There was; however, a sudden rise in PPO gene expression at the highest concentrations of BTH and BABA. The gene expression in BTH-3 and BABA-3; therefore, increased by 1.43 and 2.72 times to the control respectively (Figure 1B). The PPO gene expression levels in BTH and BABA treatment groups were in the order BTH-3 > BTH-1 > BTH-2, and BABA-3 > BABA-1 > BABA-2 respectively (Figure 1B). This result suggests that the effect of elicitor treatment on PPO gene is more complicated.

### 2.2. Effect of BTH and BABA on PPO and PAL Activities of Tea Plant

**Figure 2:**
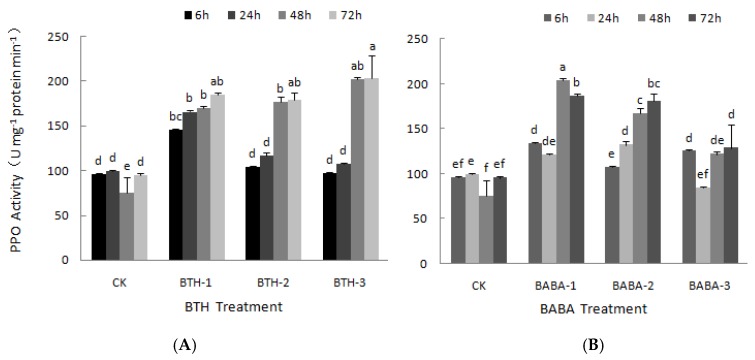
PPO activities of BTH and BABA treatments. (**A**) Changes in PPO activity of BTH treatment. (**B**) Changes in PPO activity of BABA treatment. Note: BTH-1 (25 mg/L), BTH-2 (50 mg/L), BTH-3 (75 mg/L); BABA-1 (100 mg/L), BABA-2 (200 mg/L), BABA-3 (400 mg/L). Data are presented as Means ± SD. The error bars represent the standard deviation. Different lowercase letters depict significant differences.

The effect of BTH and BABA on PPO was measured at different times, from 6 to 72 h after treatments (Figure 2). There was a steady increase in PPO activity over time with application of BTH-1 treatment; however, there was no significant difference in the activity (Figure 2A). The application of BTH-2 resulted in increase in PPO activity over time. There was a sharp increase in PPO activity by 51.59% from 24 to 48 h after treatments application, and then followed by a very narrow increase at 72 h (Figure 2A). This; therefore, resulted in a significant difference in PPO activity over time. The effect of BTH-3 on PPO was not different from that of BTH-2, there was no obvious difference in PPO activity at 48 and 72 h, yet the PPO activity increased by 88.24% from 24 to 48 h after BTH-3 application, which also in general led to a significant difference in PPO activity over time with the application of BTH-3 treatment (Figure 2A). These results; therefore, suggest that the effects of BTH on PPO activity, at various treatment concentrations, is largely dependent on time of exposure. However, at any given time (6, 24, 48 and 72 h), tea leaf PPO activities in BTH treatments increased above their corresponding control treatments.

On the other side, the effect of BABA treatment on PPO did not follow any pattern (Figure 2B). Unlike BABA-2 treatment which resulted in a steady increase in PPO activity with 6 and 72 h recording the lowest and highest PPO activity respectively, application of both BABA-1 and BABA-3 yielded an irregular pattern of PPO activity (Figure 2B). Tea leaf PPO activity decreased at 24 h and increased sharply by 68.78% at 48 h with BABA-1 treatment. However, at 72 h, the PPO activity decreased again, but slightly (Figure 2B). In the case of BABA-3, PPO activity decreased at 24 h after treatments. There was a sharp recovery in PPO activity where a stabilized increased pattern was observed from 24 to 72 h after BABA-3 treatment (Figure 2B). However, apart from 24 h after BABA-3 treatment, leaf PPO activity was higher in all other BABA treatments compared with their respective control treatments at any given time.

**Figure 3:**
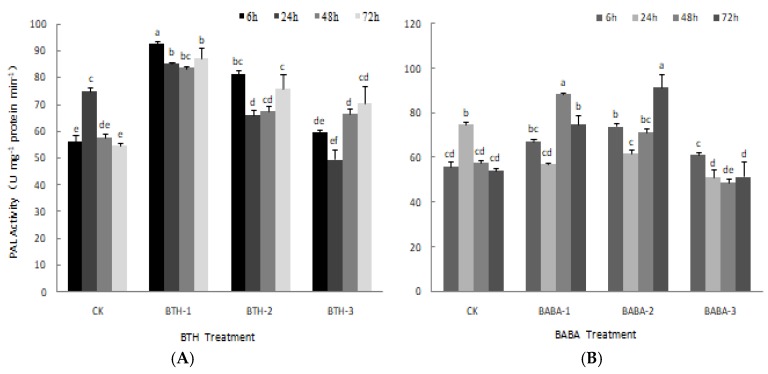
PAL activities of BTH and BABA treatments. (**A**) Changes in PAL activity of BTH treatment. (**B**) Changes in PAL activity of BABA treatment. Note: BTH-1 (25 mg/L), BTH-2 (50 mg/L), BTH-3 (75 mg/L); BABA-1 (100 mg/L), BABA-2 (200 mg/L), BABA-3 (400 mg/L). Data are presented as Means ± SD. The error bars represent the standard deviation. Different lowercase letters depict significant differences.

The effect of BTH and BABA on PAL activity is shown in Figure 3. BTH treatments, except for BTH-2 at 24 h and BTH-3 at 24 h, yielded in higher PAL activity compared with their corresponding control treatments (Figure 3A). However, the PAL activity in the experimental treatments did not follow same trend as that of the control treatment. PAL activity decreased steadily from 6 to 48 h and increased at 72 h after BTH-1 treatment. There was; however, no significant difference in PAL activity over time. The effects of BTH-2 and BTH-3 application on PAL activity followed the same trend. Both treatments resulted in a sudden decrease in PAL activity from 6 to 24 h after application, and increased steadily, thereafter (Figure 3A).

The effect of BABA treatments on PAL activity did not follow similar trend as the control experiment. PAL activity decreased with all BABA treatments from 6 to 24 h after application (Figure 3B). At 48 h, PAL activity increased with different margins with BABA-1 and BABA-2, but decreased with BABA-3 application. Similarly, at 72 h, PAL activity decreased with BABA-3 application but increased at different margins with both BABA-1 and BABA-2 (Figure 3B).

### 2.3. Effect of BTH and BABA on Growth of Geometrid Larvae

The effects of BTH and BABA treatments on survival rate, pupation rate and emergence rate are shown in Table 1. The survival rate and pupation rate were inversely proportional to concentration of BTH, that is, as BTH concentration increased, larvae survival and pupation rate decreased. Survival rate ranged from 77.78 to 88.89% while pupation rate ranged from 64.44 to 75.56%.There was no significant difference in BTH-1 and BTH-2, but both survival rate and pupation rate decreased significantly with BTH and BABA application (Table 1). Rate of emergence of larvae increased when BTH concentration was doubled from 25 to 50 mg/L, and then recorded a huge decrease (13.56%) as BTH concentration reached its peak (75 mg/L). Unlike with BTH treatments, survival rate and pupation rate decreased with application of 200 mg/L BABA, and then increased at 400 mg/L BABA treatment (Table 1). On the contrary, emergence rate showed no significant increase as the rate was similar (35.26 and 35.48%) for both BABA-1 and BABA-2, and decreased sharply (15.15%) at BABA-3. Survival rate, pupation rate and emergence rate ranged from 82.22 to 88.89%, 68.89 to 75.56% and 15.15 to 35.48% respectively with BABA treatments (Table 1). In general, there was no significant difference in BTH-1, BABA-1 and the control treatments on survival and pupation rates. There was; however, a significant difference in BTH-3, BABA-2 treatments and the control. Therefore, the best combination to inhibit growth of geometrid larvae was 75 mg/L BTH and 200 mg/L BABA.

**Figure 4:**
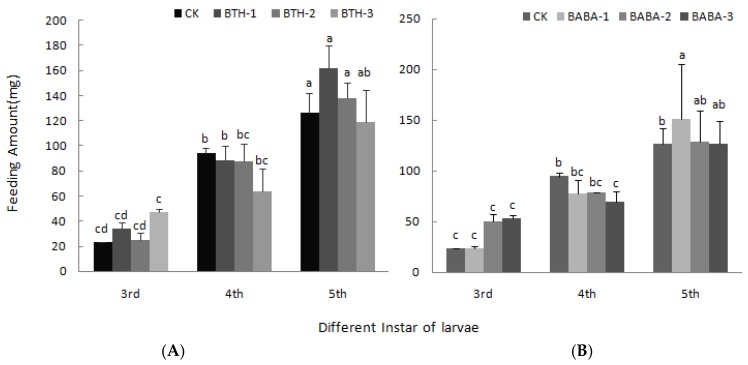
Effect of BTH and BABA on feeding amount of larvae. (**A**) Feeding amount of larvae of BTH treatment. (**B**) Feeding amount of larvae of BABA treatment. Note: BTH-1 (25 mg/L), BTH-2 (50 mg/L), BTH-3 (75 mg/L); BABA-1 (100 mg/L), BABA-2 (200 mg/L), BABA-3 (400 mg/L). Data are presented as Means ± SD. The error bars represent the standard deviation. Different lowercase letters depict significant differences.

Figure 4 shows the leaf feeding rate of larvae upon elicitor treatment. It was observed that rate of leaf feeding increased first in 3rd instar larvae, but decreased by 31.31 and 28.83% in BTH-3 and BABA-3 treatments respectively in the 4th instar larvae. Moreover, leaf feeding rate of 5th instar larvae increased in both BTH and BABA treatments relative to the control; however, there was no significant difference among treatment concentrations.

**Figure 5:**
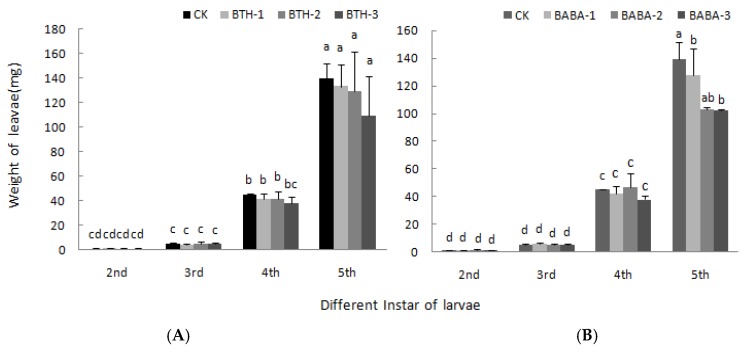
Effect of BTH and BABA on the weight of larvae. (**A**) The weight of larvae of BTH treatment. (**B**) The weight of larvae of BABA treatment. Note: BTH-1 (25 mg/L), BTH-2 (50 mg/L), BTH-3 (75 mg/L); BABA-1 (100 mg/L), BABA-2 (200 mg/L), BABA-3 (400 mg/L). Data are presented as Means ± SD. The error bars represent the standard deviation. Different lowercase letters depict significant differences.

The effect of BTH and BABA on weight of larvae is shown in Figure 5. Body weight of larvae was directly proportional to level of instar larvae, but was not affected by BTH and BABA treatments. However, at 5th instar larvae stage, body weight decreased by 22.56% when fed with BTH-3 treated leaves (Figure 5A), and also decreased by 23.85 and 26.80% when fed with BABA-1 and BABA-2 respectively (Figure 5B), relative to the control treatment. There was; therefore, no significant difference in the mean body weight of different instar larvae fed with leaves treated with various concentrations of BTH and BABA (Figure 5).

### 2.4. Effect of BTH and BABA on Midgut Proteinase Activity of Geometrid Larvae

**Figure 6:**
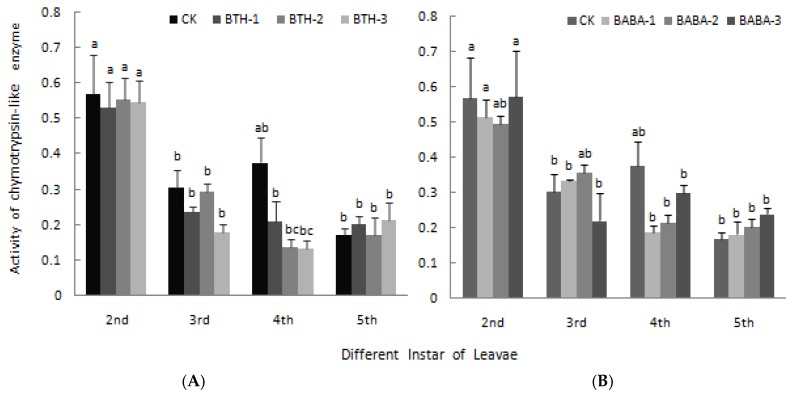
Effect of BTH and BABA on chymotrypsin-like enzyme activity. (**A**) Changes in chymotrypsin-like enzyme activity of BTH treatment. (**B**) Changes in chymotrypsin-like enzyme activity of BABA treatment. Note: BTH-1 (25 mg/L), BTH-2 (50 mg/L), BTH-3 (75 mg/L); BABA-1 (100 mg/L), BABA-2 (200 mg/L), BABA-3 (400 mg/L). Data are presented as Means ± SD. The error bars represent the standard deviation. Different lowercase letters depict significant differences.

The chymotrypsin-like enzyme activity in midgut of larvae among the different treatments as well as the control is shown in Figure 6. In the treatment groups, chymotrypsin-like enzyme activity decreased as the larvae transformed from 2nd instar stage to 4th instar stage, and increased again at 5th instar stage with BTH-2 and BTH-3 application (Figure 6A). At 2nd, 3rd and 4th instar stage, chymotrypsin-like enzyme activity generally decreased below the control treatment; however, a reverse observation was made at 5th instar stage, except for BTH-2 treatment which recorded a slight decrease in chymotrypsin-like enzyme activity. There was; therefore, no significant difference in chymotrypsin-like enzyme activity in midgut of larvae at the various larvae instar stages when treated with BTH treatments (Figure 6A). Just like BTH, BABA treatments application at different concentrations did not have any significant effect on chymotrypsin-like enzyme activity in midgut of larvae (Figure 6B).

**Figure 7:**
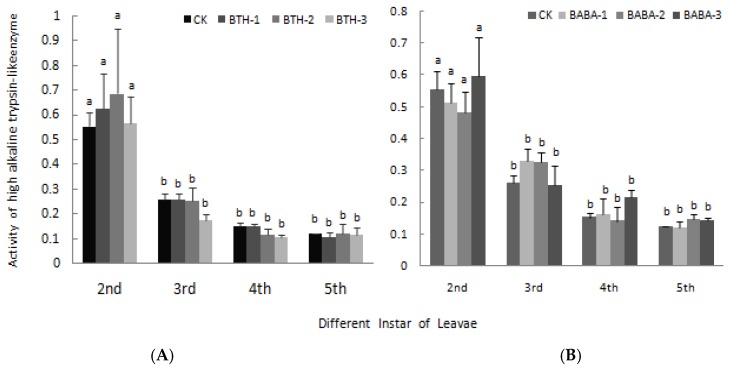
Effect of BTH and BABA on high alkaline trypsin-like enzyme activity. (**A**) Changes in high alkaline trypsin-like enzyme activity of BTH treatment. (**B**) Changes in high alkaline trypsin-like enzyme activity of BABA treatment. Note: BTH-1 (25 mg/L), BTH-2 (50 mg/L), BTH-3 (75 mg/L); BABA-1 (100 mg/L), BABA-2 (200 mg/L), BABA-3 (400 mg/L). Data are presented as Means ± SD. The error bars represent the standard deviation. Different lowercase letters depict significant differences.

**Figure 8:**
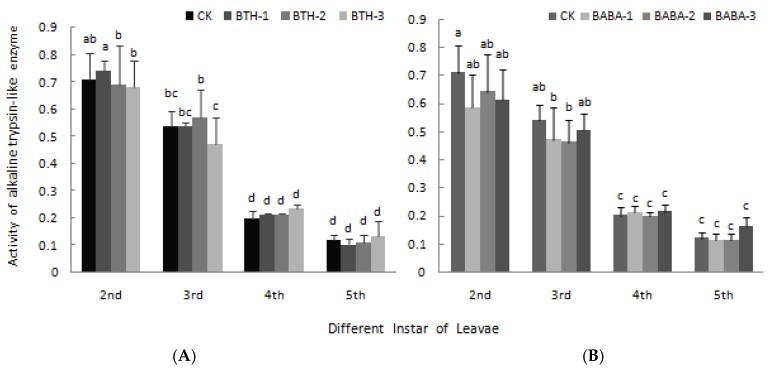
Effect of BTH and BABA on low alkaline trypsin-like enzyme activity. (**A**) Changes in low alkaline trypsin-like enzyme activity of BTH treatment. (**B**) Changes in low alkaline trypsin-like enzyme activity of BABA treatment. Note: BTH-1 (25 mg/L), BTH-2 (50 mg/L), BTH-3 (75 mg/L); BABA-1 (100 mg/L), BABA-2 (200 mg/L), BABA-3 (400 mg/L). Data are presented as Means ± SD. The error bars represent the standard deviation. Different lowercase letters depict significant differences.

The effects of BTH and BABA on high and low alkaline trypsin-like enzyme activity are presented in Figure 7 and Figure 8 respectively. BTH and BABA application did not result in any significant difference in high alkaline trypsin-like enzyme activity. However, high alkaline trypsin-like enzyme activity was high in 2nd instar larvae and low in 5th instar larvae in both BTH and BABA treatments, except for BABA-2 in 5th instar larvae (Figure 7). Generally, application of BTH and BABA treatments at various concentrations did not affect low alkaline trypsin-like enzyme activity, except for the individual data at 2nd and 3rd instar stages (Figure 8). However, low alkaline trypsin-like enzyme activity was dependent on stage of instar larvae, in the order 2^nd^ > 3^rd^ > 4^th^ > 5^th^ for both BTH and BABA treatments (Figure 8). There was; therefore, no significant difference in both high alkaline trypsin-like enzyme activity and low alkaline trypsin-like enzyme activity upon BTH and BABA treatments (Figure 7 and Figure 8).

### 2.5. Effect of BTH and BABA on Geometrid Larvae Development

The development duration of larvae instar fed with the tea leaves treated with elicitors increased compared to the control groups (Table 2). With BABA treatment, the development duration of larvae delayed to 26.33 d, while development duration delayed to 23.33 d with BTH treatments (Table 2). The development duration in both treatments was more than in the control experiment (15.33 d). It could; therefore, be suggested that elicitor treatments delayed geometrid larvae development.

## 3. Discussion

With their special function, coupled with their advantages of non-toxicity and no pollution, benzothiadiazole and β-aminobutyric acid have been widely adopted to induce pathogen resistance in crops such as tobacco, wheat and maize. It was therefore reported that foliar application of elicitors at various concentrations actually induced systemic acquired resistance of these crops to pathogens [19,20]. Elicitors have also been reported to reduce insect infestation in tomato plant and Brassicaceae [21,22]. Plant induced resistance consists of direct and indirect mechanisms. Direct resistance, which is the most important, is mainly based on the biochemical change in plant tissue, and directly disturbs the digesting system of insect, leading to impairment in growth and development. PAL, PPO and POD are the key enzymes involved in the direct resistance to herbivores in tea plant [12,13,23,24]. PAL can adjust the synthesis and accumulation of plant phenol compounds, as high content of phenol compounds in general are of disadvantage to herbivores. PPO, POD and other isozymes can affect the growth and development of plant pathogens; moreover, the latter can strongly combine with proteins, including all kinds of midget enzymes of insects, which may lead to physiological disorder and subsequent developmental delay in herbivores [18]. In present study the activities of PPO and PAL, as well as the expression of two genes related to the enzymes in the tea plant treated with BTH and BABA were investigated. The results in this study suggested that PAL and PPO were activated upon BTH and BABA treatments; however, this was highly dependent on the concentration of treatment and time. PAL and PPO activation resulted in corresponding reduction in survival rate, pupation rate and emergence rate of geometrid larvae. This also resulted in delay in developmental process of the instar larvae. The delay in development duration of larvae is directly proportional to the concentration of elicitor applied. Elicitor application resulted in the reduction in weight of geometrid larvae. Several researches have reported that PPO and PAL are induced by elicitors, and could act as resistance related indicative enzymes [25,26]. Also, in other studies, elicitors such as JA and chitosan were found to be effective in inducing plant acquired immunity [27]. These reports are in conformity with the results in the present study.

Identification of insect infestation usually occurs when avirulence (Avr) gene products, secreted by insects, interact with the product of a plant resistance (R) gene [28]. A putative binding of these two partner genes results in activation of a signal transduction cascade, which leads to subsequent activation of a variety of plant defense responses and related genes leading to active resistance of the plant to the insects [28].PAL gene is a key gene in phenylpropanoid pathway, which is essential for the biosynthesis of multiple phenolic compounds. The secondary metabolites play a major role in insect resistance [29]. In an experiment conducted by Wang et al., differential transcriptome analysis on defense responses of tea plant to *Ectropis obliqua* revealed that most expressed genes in phenylpropanoid pathway were upregulated, including PAL [30]. Consistent with this report, qRT-PCR revealed that gene expression of PAL and PPO up-regulated with corresponding rise in enzyme activity. At higher concentration of elicitor treatment, relative expression of PAL and PPO genes was up-regulated, relative to the control. However, the down-regulation of PPO gene expression at lower concentrations did not correspond to the activity change of PPO. This could be attributed to the fact that PPO is a multi-gene code enzyme whose activity is largely dependent on the kind of functional gene regulated by elicitor [31]. In an experiment conducted on two kinds of larchs (*Larixgmelinii* (Rupr.) Kuzen.), Xu et al. reported that an activation of PPO and POD, as well as their related genes, by exogenous jasmonic acid result in direct and indirect resistance to Gypsy moth (*Lymantria dispar* L.) [32], and this compares favourably with the result in the present study. It could be inferred that benzothiadiazole and β-aminobutyric acid help resist the attacks of *Ectropis obliqua* by increasing the expression of PAL and PPO genes and increasing the biosynthesis of polyphenols.

When a plant is primed, the priming stimulus information is stored, ultimately until exposure to a triggered stimulus, and can last for more than 8 weeks [33]. This effect is known as the “memory” of plant defense [34]. In the present study, it was observed that activities of PPO and PAL of primed plants generally increased above the control, reaching its optimal at 72 h, except for the PAL activity of BABA-3. Contrarily, Ren et al. reported an optimal PPO activity at 10 h followed by a steady decline as exposure time increased beyond 10 h [8]. The discrepancies in these results could be attributed to the source and dose of elicitor employed in both studies. While Ren et al. used extracts from endophytic fungus *Cunninghamella sp*. (AL4) at lower concentrations (up to 40 mgL^−1^), the present study employed synthetic elicitor at a relatively higher concentrations.

Analysis of proteinase activities present in larvae midgut extracts from several lepidopteran insects revealed that serine proteinases, elastase, trypsin and chymotrypsin are the major proteolytic activities detected [35]. According to Guo et al., chymotrypsin-like enzyme, high alkaline trypsin-like enzyme and low alkaline trypsin-like enzyme coordinate each other in a balanced proportion to give insects the ability to deduce the affection of phenol-protein complex [36]. High alkaline trypsin-like enzyme and low alkaline trypsin-like enzyme are responsible for toxin detracting while chymotrypsin-like enzyme often correlate with food protein digestion as well as toxin detraction [37]. In the present study, analysis of effect of BTH and BABA on the hydrolysis of synthetic substrates revealed that chymotrypsin-like enzyme activity present in larval midgut extracts was not significantly inhibited by BABA and BTH. Tamayo et al. reported that chymotrypsin-like activities from the larval midgut of *Spodoptera littoralis* was significantly inhibited by maize proteinase inhibitor (MPI). In their study, low concentrations of MPI inhibitor effectively inhibit elastase and chymotrypsin-like activities present in the midgut extract of S. *littoralis* larvae [35]. However, their report is not consistent with the result in this study. It was observed, in the present study, that proteinase activity generally declined as TG mature, even though the trend was not consistent. The imbalanced midget proteinase activity and its resultant digestive disorder may partly explain the growth decline in *Ectropis obliqua* larvae fed with elicitor-treated tea leaves [38,39].

Researches have shown that BTH and BABA induced plant resistance to pathogens and insects is not based on its direct toxicity [40], but mainly through callose formation and SA, ABA signaling pathway [41]. BABA induced callose deposition occurs at the sites of pathogen penetration; thus, preventing spread of the pathogen [42]. According to Will et al. [43,44] and Tjallingii [43,44], callose is also involved in plant phloem sealing mechanisms, which could confer plant resistance to aphids. BABA acts by potentiation of a normally under-expressed pathway [45]. In a study conducted by Maet al., it was reported that 0.5 mmol/L BTH could induce systemic resistance in cucumber to *Cladosporium cucumerinum* infection [46]. Benzothiadiazole (BTH) enhanced the accumulation of soluble and cell-wall-bound phenolics in strawberry leaves and also improved the resistance to powdery mildew infection under greenhouse conditions [47]. Also BABA applied as a soil drench reduced aphid performance. When applied as a foliar spray, BABA induced the formation of pinpoint necrotic spots which are considered to be involved in systemic acquired resistance [48].Consistent to earlier reports, the results in the present study; therefore, indicated that BTH and BABA could induce insect resistance ability in tea plant.

Upon perception of elicitor, plants undergo activation of signal transduction pathways which generally results in the production of active oxygen species (AOS), deposition of callose, reinforcement of plant cell wall associated with phenyl propanoid compounds, synthesis of defense enzymes, phytoalexin biosynthesis and the accumulation of pathogenesis-related (PR) proteins, and eventually conferring pest resistance ability to plants [49]. This mechanism could possibly explain the results in the present study.

## 4. Materials and Methods

### 4.1. Plant and Chemicals

The study was conducted in a 10-year-old ‘Longjing43’ tea cultivar plantation at tea garden of Sun Yat-sen Mausoleum scenic spot in Nanjing, Jiangsu Province, China. BABA and BTH were purchased from Gray Asia Chemical Co. Ltd. (Chengdu, China). The chemicals were dissolved in 95% ethanol, and then diluted with water to obtain final concentrations of 25, 50 and 75 mg/L BTH solutions (marked as BTH-1, BTH-2 and BTH-3, respectively), and 100, 200 and 400 mg/L BABA solutions (marked as BABA-1, BABA-2 and BABA-3, respectively). Twenty-one experimental plots each of 15 m^2^ representing various levels of BABA, BTH and control treatments were established. Each treatment was replicated three times. For each treatment plot, 1 L of elicitor was sprayed on the tea leaves, while the control plot received no application.

### 4.2. Growth Environment of Ectropis obliqua

Instar larvae of tea geometrid were collected from the tea garden in Sun Yat-sen Mausoleum scenic spot, Nanjing, China and kept in a cuboid box (enclosed with nylon) under room temperature. The insects were fed with ‘Longjing 43’ tea leaves and allowed to reproduce. The second generation tea geometrid larvae were collected and kept in a light incubator with 25 ± 1 °C, 75% RH and a 16/8 h photoperiod until 2nd instar stage, then were selected for the experiment.

### 4.3. Analysis of PPO and PAL Activities

Tea shoots were picked at 6, 24, 48 and 72 h after treatments respectively and placed in liquid nitrogen for physiology and biochemical analysis according to the methods described by Yang et al., with modifications [24]. Fresh leaves (0.5 g) were used for crude enzyme extraction [14]. For PPO analysis 3 mL reaction mixture (consisting of pH 7.0 phosphate buffer solution, 0.1%Pro and 0.1% pyrocatechol at a volume ratio of 10:2:3) incubated with 1 mL natural enzyme was used, while for the assessment of PAL activities, 1 mL natural enzyme was added to the 2 mL reaction mixture (containing 1 mL pH 7.0 phosphate buffer solution and 1 mL 0.02 mol·L^−1^ Phe). Both mixtures were incubated at 37 °C for 30 min, and then stopped by 0.5 mL 1 mol·L^−1^ hydrochloric acid. Content of soluble protein was measured using the approach introduced by Bradford et al. based on the bovine serum albumin protein [50]. The unit for quantifying enzyme activity was described individually as the absorbance by 0.1 for PPO and 0.01 for PAL and expressed as U mg^−1^ protein min^−1^.

### 4.4. Biological Activity of Geometrid Larvae

After 72 h of the chemical treatments, tea leaves were randomly collected from the experimental plots at two days interval, placed in a plastic box and stored at 4 °C. The leaves were cut into 1 cm diameter discs and fed to tea geometrid larvae. The experiment was conducted in a petri dish (9 cm in diameter) [13,14]. Each treatment concentration including the control represented a petri dish, and was replicated three times, making a total of 21 petri dishes. Each petri dish also contained three 2nd instar larvae of tea geometrid. A known weight of 8 pieces of 1 cm diameter discs of the treated leaves were fed to the larvae in the corresponding petri dishes until the larvae reached the pupa stage. From start of the experiment, the test leaf discs were replaced after every 48 h and then every 24 h from the eighth day, with corresponding data taken on weight of remaining leaf disc and body weight of larvae. After the eighth day pupation rate, feathering rate and survival rate of pupa were also recorded every 48 h. The development duration of tea geometrid was calculated from when feeding of larvae began till pupa formation.

### 4.5. Analysis of Midgut Proteinase Activities of Geometrid Larvae

The larvae were weighed, homogenized in liquid nitrogen and centrifuged at 15,000 *g*, 4 °C for 20 min. The supernatant was used for the midgut proteinase activity determination following the protocol described by Gui et al. with modifications [13]. The activities of the low and high alkaline trypsin-like enzyme and chymotrypsin-like enzyme were also measured.

### 4.6. Analysis of PAL and PPO Genes Expression

Fresh leaves were collected after 72 h of chemical treatments. Total RNA was extracted according to the method described by Fu [51]. The total RNA was then reverse-transcribed into first strand cDNA using 20 μL Super RT cDNA Kit (BioTeke, Beijing, China) with 0.2 μg RNA, 1 μL oligo dT, 4 μL dNTP mixture, 4 μL 5× first-strand buffer, 1 μL M-MLV reverse transcriptase and 1 μL RNase inhibitor. The transcription process included two steps 50 °C for 40 min and 70 °C for 10 min. PAL and PPO gene primers (Table 3) for the qRT-PCR analysis were designed and synthesized by Invitrogen Co. Ltd. β-actin was used as the reference gene to analyze the property of PAL and PPO genes quantitatively. The PCR amplification condition used involved 40 cycles of 95 °C for 60 s, 95 °C for 10 s, 60 °C for 10 s and 72 °C for 30 s. The relative abundance was noted using the 2^−∆∆CT^ method. Each measurement was repeated 3 times.

### 4.7. Statistical Analysis

All experiments were repeated 3 times. The statistical analysis was performed with SPSS version 17.0 (SPSS Inc., Chicago, IL, USA). Data were analyzed by one-way analysis of variance (ANOVA).

## 5. Conclusions

In this study, foliar application of benzothiadiazole (BTH) and β-aminobutyric acid (BABA) ultimately inhibited growth, development and performance of geometrid larvae as characterized by decreased body weight, prolonged development duration, and lower rates of survival, pupation and emergence of tea geometrid larvae relative to the control. Analysis of hydrolysis of synthetic substrates also revealed that chymotrypsin-like enzyme activity present in larval midgut extracts was not significantly inhibited by BTH and BABA. Moreover, proteinase activity was found to be inversely proportional to the age of tea geometrid. The optimal effect of BTH and BABA was achieved at BTH-3 (75 mg/L) and BABA-3 (400 mg/L) respectively. This; therefore, suggests that BTH and BABA, at higher concentration, have the potential to induce systemic resistance in tea plant to *Ectropis obliqua*. The results in this study would contribute to the knowledge base of BTH- and BABA-mediated plant resistance to insects.

## Figures and Tables

**Table 1:** Effect of BTH and BABA on the rates of survival, pupation and emergence of instar larvae.

Treatment	Survival Rate (%)	Pupation Rate (%)	Emergence Rate (%)
CK	95.56 ± 3.85 ^a^	82.22 ± 3.85 ^a^	43.38 ± 0.60 ^a^
BTH-1	88.89 ± 1.02 ^a^	75.56 ± 1.02 ^a^	26.32 ± 0.07 ^a,b^
BTH-2	86.67 ± 0 ^a^	73.33 ± 0 ^b^	39.39 ± 0.52 ^a^
BTH-3	77.78 ± 1.02 ^b^	64.44 ± 1.02 ^b^	13.56 ± 0.42 ^b^
BABA-1	88.89 ± 1.39 ^a^	75.56 ± 1.39 ^a^	35.26 ± 0.57 ^a,b^
BABA-2	82.22 ± 1.39 ^b^	68.89 ± 1.39 ^b^	35.48 ± 0.24 ^a,b^
BABA-3	84.44 ± 0.38 ^b^	71.11 ± 10.38 ^b^	15.15 ± 0.14 ^b^

Means within a row indicated by different lowercase letters are significantly different (LSD test, *p* < 0.05).

**Table 2:** Effect of BTH and BABA on *Ectropis obliqua* larvae development (in days).

Treatment	3rd Instar	4th Instar	5th Instar	Development Duration
CK	2.67 ± 0.58 ^a^	2.00 ± 1.00 ^a^	2.33 ± 0.58 ^a,b^	15.33 ± 1.53 ^a^
BTH-1	3.33 ± 0.58 ^a,b^	3.33 ± 0. 58 ^a,b,c^	2.67 ± 0.58 ^a,b^	19.00 ± 2.00 ^a,b^
BTH-2	2.67 ± 1.15 ^a^	3.00 ± 1.15 ^a,b,c^	2.33 ± 0.58^a,b^	17.33 ± 1.53 ^a^
BTH-3	3.67 ± 0.58 ^a,b,c^	4.33 ± 0.58 ^c^	3.33 ± 0.58 ^b^	23.33 ± 2.52 ^c,d^
BABA-1	2.67 ± 0.58 ^a^	2.67 ± 0.58 ^a,b^	2.00 ± 0 ^a^	19.00 ± 2.00 ^a,b^
BABA-2	4.00 ± 0 ^b,c^	3.33 ± 0.58 ^a,b,c^	2.33 ± 0.58 ^a,b^	21.33 ± 1.15 ^b,c^
BABA-3	4.67 ± 0.58 ^c^	4.00 ± 0 ^b,c^	4.33 ± 0.58 ^c^	26.33 ± 3.21 ^d^

Means within a row indicated by different lowercase letters are significantly different (LSD test, *p* < 0.05).

**Table 3:** Primers used for qRT-PCR analysis for PAL and PPO gene expression.

Gene Name	Forward Primer	Reverse Primer
PAL	GCGCGTTCTAACTAACTATGGG	TAGTGGATAAGACCGGCATTC
PPO	GGCTCTTCTTTCCGTTCC	CTAGATTCGGCTGGGTGC
β-actin	GCCATCTTTGATTGGAATGG	GGTGCCACAACCTTGATCTT
